# Validation of a self-report questionnaire for periodontitis in a Japanese population

**DOI:** 10.1038/s41598-021-93965-4

**Published:** 2021-07-23

**Authors:** Masanori Iwasaki, Michihiko Usui, Wataru Ariyoshi, Keisuke Nakashima, Yoshie Nagai-Yoshioka, Maki Inoue, Kaoru Kobayashi, Wenche S. Borgnakke, George W. Taylor, Tatsuji Nishihara

**Affiliations:** 1 Tokyo Metropolitan Institute of Gerontology, 35-2 Sakae-cho, Itabashi-Ku, Tokyo, 173-0015 Japan; 2 Division of Periodontology, Kyushu Dental University, Kitakyushu, Japan; 3 Division of Infections and Molecular Biology, Kyushu Dental University, Kitakyushu, Japan; 4 Dental Center for Regional Medical Survey, Kyushu Dental University, Kitakyushu, Japan; 5 Graduate School of Dentistry, MSc Program, Kyushu Dental University, Kitakyushu, Japan; 6 Department of Periodontics and Oral Medicine, University of Michigan School of Dentistry, Ann Arbor, MI USA; 7 Division of Oral Epidemiology and Dental Public Health, Department of Preventive and Restorative Dental Sciences, UCSF School of Dentistry, San Francisco, CA USA

**Keywords:** Epidemiology, Periodontitis

## Abstract

We aimed to assess the validity of the self-report questionnaire for periodontitis in a Japanese population. A Japanese 9-item self-report questionnaire, developed by translating English-version questions that were used to detect periodontitis, was validated against full-mouth clinically-assessed periodontitis in 949 Japanese adults (average age = 43.2 years). Multivariable logistic regression modeling was used to calculate the area under the receiver operating characteristic curve (AUC), wherein the periodontitis case definition of the Centers for Disease Control and Prevention/American Academy of Periodontology was considered the gold standard. Severe, moderate, and mild periodontitis were identified in 6.2%, 30.0%, and 6.7% of the study population, respectively. Self-reported oral health questions combined with socio-demographic and health-related variables had an AUC > 0.70 (range, 0.71–0.87) for any periodontitis category. Four oral health questions (“have gum disease,” “loose tooth,” “lost bone,” and “bleeding gums”) were selected in the parsimonious model for severe periodontitis. The periodontitis screening score generated by the responses to these four questions had an AUC, sensitivity, and specificity of 0.82, 73.1%, and 74.3%, respectively, where the cut-off was set at 2 points. In conclusion, a locally adapted version of the self-report questionnaire had an acceptable diagnostic capacity for the detection of periodontitis in this study population.

## Introduction

Periodontitis is a chronic bacterial infection that results in an inflammatory destruction of the connective tissue and bone supporting the teeth^1^. In Japan, periodontitis is common, with an estimated prevalence of 49.4%^2^. It is the leading cause of tooth loss in adults^3^. Tooth loss leads to decreased oral function and quality of life^4,5^. Furthermore, periodontitis is associated with systemic diseases (such as diabetes mellitus, chronic kidney disease, and cardiovascular disease)^6–8^, increased medical care cost^9^, and mortality^10^. Considering its impact on individuals and society, periodontitis prevalence reduction among adults is one of the targets of the second term of Health Japan 21 serving as a national health promotion measure^11^. Epidemiological surveillance of periodontitis is important to monitor its prevalence, and surveillance results can be used for the planning and evaluation of health promotion activities targeting periodontitis.

In population surveillance and epidemiologic research, a full-mouth periodontal examination (FMPE) is considered the gold standard; it entails measuring the periodontal pockets at six sites (mesio-buccal, mid-buccal, disto-buccal, mesio-lingual, mid-lingual, and disto-lingual) on every tooth^12^. However, many resources, including work force (trained and calibrated examiners), equipment, and time, are required for clinical periodontal examinations. Thus, population level surveillance of periodontitis is challenging. This is mirrored by the current situation in Japan where the number of individuals participating in the Survey of Dental Diseases in Japan (SDD), is decreasing^2^. In addition, due to the limitation of time and resources, a partial-mouth periodontal examination (PMPE), not FMPE, was performed in the SDD. A PMPE is reported to have a low validity for surveillance and research^13,14^. This low validity is due to the fact that periodontitis is not symmetrically distributed in the mouth, preventing valid extrapolation from the PMPE collected data. Furthermore, according to a recent survey conducted by the Japan Dental Association^15^, approximately 70% of the surveyed adults reported that they did not visit the dentist regularly. Overall, periodontal surveillance is not commonly performed in Japan, and many Japanese adults miss early diagnosis of periodontal diseases and appropriate treatment.

The self-report questionnaire is a potential alternative strategy. The main advantage of the self-report questionnaire is that it is a relatively simple way to collect data from many individuals in a time- and cost-effective manner. An 8-item questionnaire was proposed in a collaboration between the Centers for Disease Control and Prevention (CDC) and the American Academy of Periodontology (AAP)^12,16–18^. Starting with the 2009–2010 National Health and Nutrition Examination Survey (NHANES) cycle, the eight questions were included in the protocol and will serve to monitor the prevalence of periodontitis in American dentate adults aged > 30 years over time, instead of a clinical periodontal examination. This CDC/AAP questionnaire has been validated in an American population^12,17,19^. The Spanish, French, Dutch, and Korean versions of the CDC/AAP questionnaire have also been validated^20–23^. Researchers also proposed a user-friendly score for periodontitis screening based on their responses to the questions selected from the 8-item questionnaire along with other variables related to periodontitis^20,22^. Overall, the most widely used questionnaire is that of the CDC/AAP^20^.

Based on these findings, the current study was designed with the aims of (1) developing a self-report questionnaire on periodontitis by translating existing English-version questions, including those proposed by the CDC/AAP, with a local modification to the Japanese version and (2) assessing the validity of the self-report questionnaire in a Japanese population. Our secondary aim was to develop a screening score for severe periodontitis based on the self-reported oral health items.

## Methods

### Development of the Japanese version of the self-report questionnaire

We developed a draft of the 9-item questionnaire (the original English version of the questionnaire; EV1) based on the 8-item CDC/AAP questionnaire and one additional item on gingival bleeding (Table S1). A recent narrative review on the self-report of periodontitis in a Japanese population^24^ revealed that the question on bleeding gums was the most used self-reported item to assess periodontal health in this population. In addition, a previous study reported that the question on bleeding gums is useful for screening for periodontitis among Japanese male workers^25^.

According to a previous study^26^, the adaptation process of the original English-version questionnaire items to the Japanese version consisted of the following six stages:

Stage 1.: The translation of the EV1 into Japanese was performed by one independent native English-speaking certified translator (T1) and two other independent native-speaking Japanese specialists in periodontology (T2 and T3).
Stage 2.: A preliminary version (the first Japanese version of the questionnaire; JV1) was synthesized by combining the best cultural and clinical translation of each item by T1, T2, and T3 together. This process was performed by an expert committee (composed of M. Iwasaki and three other specialists [Y.M., H.O., and A.Y., who are not authors of this manuscript] in oral epidemiology and periodontology).
Stage 3.: Two independent native English-speaking certified translators, back-translated the JV1 from Japanese to American English and the back-translated version (EV2) was created.
Stage 4.: WBS and GWT, who participated in creating the original CDC/AAP questionnaire^12^ ensured that the EV2 reflected the same item content (literal, conceptual, and semantic equivalence) as the EV1. At this stage, several items had to go through the earlier stages repeatedly until WBS and GWT provided a final certification. This process was used to revise the JV1 and build the Japanese version 2 (JV2).
Stage 5.: JV2 was pre-tested to assess the clarity of the instructions and responses regarding language in a group of 100 non-professional individuals (composed of 20 men and 20 women aged between 35 and 44 years, 20 men and 20 women aged between 45 and 55 years, and 10 men and 10 women aged between 56 and 65 years). A third-party marketing research company (NEO MARKETING INC., Tokyo, Japan) recruited these 100 non-professional individuals. The 100 individuals were first asked to complete the JV2. Then, they were asked to rate each questionnaire item on a 5-point Likert scale according to the following instruction: “Please indicate the extent to which you agree or disagree with the following statement: question and responses are clear and easy to understand [1-strongly disagree, 2-disagree; 3-neutral; 4-agree; and 5-strongly agree])”. At this stage, item 3 (Have you ever had treatment for gum disease, such as scaling and root planing, sometimes called “deep cleaning”?) was found to have poor clarity (Table S2). This item was passed again through the earlier stages, and revision was made to the format, wording, and presentation of the questions. The results were used to revise the JV2 and build the Japanese version 3 (JV3).
Stage 6.: The JV3 was submitted to an expert committee to assess and elaborate on the content validity of the scale. The items in the JV3 were rated on a 4-point Likert scale (1-irrelevant, 2-little relevance, 3-relevant, and 4-extremely relevant) where 1 and 2 were considered irrelevant, while 3 and 4 were considered relevant. Several changes were made to the format, wording, and presentation of each JV3 item, until all the items were rated as relevant by all the four expert committee members. Following the discussion and agreement of the expert committee, a final version of the Japanese questionnaire (JV4) was developed (Table S1). This final version was back-translated into English (EV3) by a native English-speaking certified translator blinded to the original version. WBS and GWT confirmed that EV3 reflected the same item content (literal, conceptual, and semantic equivalence) as EV1.

### Assessment of validity of the developed Japanese questionnaire

#### Design, setting, and participants

The study population comprised a cohort of workers living in Fukuoka Prefecture, Japan. Individuals who participated in an annual health check-up at the workplace between December 2019 and March 2020 were invited to participate in the current study. In Japan, employees undergo periodical medical examinations at least once a year if their workplaces regularly employ 50 or more workers, according to the Industrial Safety and Health Act. Our validation study of the questionnaire was designed to be conducted during this periodical medical examination.

The inclusion criteria were as follows: at least 20 years of age and able to read and understand Japanese. The exclusion criteria were as follows: having less than 2 teeth and pre-diagnosed severe or terminal disease, such as advanced heart failure, end-stage kidney disease, or advanced-stage cancer.

All the study participants provided written informed consent prior to being included in the study. The present study was conducted in full accordance with the ethical principles of the Declaration of Helsinki and was approved by the Ethics Committee of Kyushu Dental University (Approval number: 19-32).

Sample size calculation was performed using R version 4.0.2 (R Foundation for Statistical Computing, https://www.r-project.org). Considering the prevalence of severe periodontitis of 5% observed in a previous study conducted at another workplace in Fukuoka Prefecture, Japan^27^, the sample size was estimated at 747, with an area under the receiver operating characteristic curve (AUC) of 0.70, corresponding to a one-sided alpha of 0.01 and a power of 95%.

#### Questionnaire examination

The questionnaire included the Japanese 9-item questions on periodontitis that was developed and administered to every participant. Data on age, sex, smoking status, physicians’ diagnosis of diabetes mellitus, and insulin or other glucose-lowering drug use, were also collected using the questionnaire. Age was categorized into < 45, 45–54, and ≥ 55 years. Smoking status was dichotomized into current smoker or not.

#### Oral health examinations

Twelve calibrated dentists, blinded to the questionnaire responses, conducted the oral health examinations. They determined the number of teeth and presence of dentures, assessed the oral hygiene status and behavior, and recorded the periodontal probing depth (PPD), gingival recession (GR), and bleeding on probing (BOP) at six sites on every tooth, except the third molars. A periodontal probe and a mouth mirror (Williams Colorvue Probe and HD Mirrors, Hu-Friedy Mfg. Co., LLC., Chicago, IL, USA) were used under sufficient artificial illumination. Then, clinical attachment loss (CAL) was calculated using the PPD and GR. In addition, the periodontal inflamed surface area (PISA) was calculated using the PPD, GR, and BOP results. The PISA quantifies the amount of inflamed periodontal tissue in square millimeters^28^. Based on a previous study^12^, the number of teeth lost was categorized into no tooth loss, 1–5 teeth lost, and ≥ 6 teeth lost.

The pre-study calibration for PPD and recession was conducted at Kyushu Dental University by examining volunteer patients. All the examiners obtained intra-examiner kappa values > 0.8 for both PPD and GR. In addition, 11 examiners obtained kappa values > 0.8 compared to the gold standard value by another examiner (M.U.). For the kappa calculations, PPD and GR values that were exactly equal or with a difference within 1 mm indicated agreement.

The oral hygiene status was assessed using the simplified oral hygiene index (OHI-S)^29^. The OHI-S ranges from 0 to 6, with higher scores indicating a poorer oral hygiene status. Data on oral health behavior, including tooth brushing frequency (≥ 2 times/day or < 2 times/day) and regular dental check-up (yes or no) were obtained through an interview during the oral health examination.

#### Health check-up record data collection

Body mass index (BMI) and serum glycated hemoglobin A1c (HbA1c) levels were obtained from the health check-up records. Overweight was defined as a BMI ≥ 25 kg/m^2^. Based on the self-reported responses and serum data, diabetes mellitus was defined according to the physician’s diagnosis and/or self-reported use of insulin or other glucose-lowering drugs and/or HbA1c ≥ 6.5%.

#### Statistical analyses

For the evaluation of the self-report questionnaire on periodontitis, the periodontitis case definition according to the CDC/AAP (CDC/AAP definition)^30^ served as the gold standard reference for the predictive validity. Following the CDC/AAP definition, severe periodontitis was defined as having ≥ 2 interproximal sites with a CAL ≥ 6 mm (not on the same tooth) and ≥ 1 interproximal site with a PPD ≥ 5 mm; moderate periodontitis was defined as having ≥ 2 interproximal sites with a CAL ≥ 4 mm (not on the same tooth) or ≥ 2 interproximal sites with a PPD ≥ 5 mm (not on the same tooth); mild periodontitis was defined as having ≥ 2 interproximal sites with a CAL ≥ 3 mm (not on the same tooth) and ≥ 2 interproximal sites with a PPD ≥ 4 mm (not on the same tooth) or one interproximal site with a PPD ≥ 5 mm; or no periodontitis, defined as the absence of mild, moderate, or severe periodontitis.

To describe the study population characteristics according to the periodontal classification, the Student’s *t*-test, Mann–Whitney *U* test, and chi-squared test were used. The Shapiro–Wilk test was used to determine whether continuous variables were normally distributed.

The association between individual self-reported questionnaire items and the periodontal classification was evaluated using the chi-squared test. Then, crude odds ratios (ORs) with 95% confidence intervals (CIs) were calculated for each of the following items using binary logistic regression analyses: (1) severe versus no + mild + moderate periodontitis, (2) moderate + severe versus no + mild periodontitis, and (3) mild + moderate + severe versus no periodontitis. According to the previous validation study^20^, we combined infrequent categories of the self-report questions, which resulted in the dichotomization of each self-reported questionnaire item, in the logistic regression analyses. Dichotomized coding (0 or 1) for each item is summarized in Table 2, where 0 or 1 in parentheses is assigned for each response. For all items except item 9, the “refused” category, was excluded from the analyses. For items 1–6, the ORs after excluding the “don’t know” responses from the analyses were also calculated.

The Kendall’s Tau-b correlation coefficient was used to assess the correlations between pair-wise dichotomized periodontitis questionnaire items.

Furthermore, we performed multivariable logistic regression analyses to predict the prevalence of periodontitis. Logistic regression analyses were performed for the participants whose responses did not include “refused” for any of the questions. Six models were constructed for each outcome variable. Model 1 included the self-reported questions, except that on bleeding gums (CDC/AAP 8-question model). Model 2 added the question on bleeding gums. Model 3 included demographic and health- related variables (age, sex, smoking status, diabetes mellitus, overweight, and tooth loss). Model 4 included a combination of self-reported oral health questions and demographic and health-related variables (Full model). Model 5 included the best significant subset of the self-reported oral health questions that were selected using the method of all possible equations (the parsimonious model, for the self-reported oral health questions). Model 6 included the best significant subset selected from the full model. The predictive validity of the models was assessed by calculating the AUC. We also calculated the sensitivity, specificity, and Bayesian information criterion. The sensitivity and specificity were based on the dichotomized classification of the predicted probability of being a case at a cut-off point selected as close as possible to the predicted prevalence of the outcome.

In a sensitivity analysis, the performance of the self-reported oral health questionnaire in detecting periodontitis was assessed against two other periodontitis outcome variables, namely, (1) severe periodontitis based on the case definition proposed by the European Federation of Periodontology (EFP) and the AAP (EFP/AAP definition)^31^, and (2) the highest quintile of PISA. Following the EFP/AAP definition, severe periodontitis was defined as having an interdental CAL ≥ 5 mm in ≥ 2 nonadjacent teeth or a buccal or oral CAL ≥ 3 mm with a PPD > 3 mm detectable in ≥ 2 teeth, and an interdental CAL at the site of the greatest loss ≥ 5 mm^31^.

Statistical analyses were carried out using Stata version 16.1 (Stata Corporation LP, College Station, TX, USA), with the level of significance (two-tailed) set at 0.05.

## Results

### Characteristics of the participants in the study assessing the validity of the developed Japanese questionnaire

Among the 1993 individuals who underwent medical check-up and met the inclusion criteria, 1045 agreed to participate in our study; 96 of these participants had missing data and were excluded from the analyses. Overall, the study population comprised 949 adults (average age = 43.2 years; age range = 20–77 years; 267 men and 682 women).

Based on the CDC/AAP periodontitis definitions, 59 participants (prevalence = 6.2%) had severe periodontitis, 285 (prevalence = 30.0%) had moderate periodontitis, and 64 (prevalence = 6.7%) had mild periodontitis.

Table 1 presents the comparison of health characteristics according to the presence and severity of periodontitis. Periodontitis was associated with poor periodontal health parameters, higher number of teeth lost, proportion of denture used, and poor oral hygiene status. In addition, periodontitis was associated with advanced age, male sex, current smoking status, overweight, and diabetes mellitus.

**Table 1:** Characteristics of the study population according to the CDC/AAP periodontitis definition.

	Total	Periodontitis category				*p*
		No	Mild	Moderate	Severe	
	N = 949	n = 541	n = 64	n = 285	n = 59	
Average PPD (mm)^b^	1.7 (1.5–2.0)	1.6 (1.4–1.8)	1.8 (1.6–2.0)	1.9 (1.6–2.3)	2.5 (2.1–2.8)	< 0.001
**Percentage of sites with PPD**^**b**^						
≥ 3 mm	7.7 (3.1–19.8)	4.2 (1.2–8.6)	13.0 (6.3–23.8)	17.9 (8.6–31.5)	37.0 (23.2–52.6)	< 0.001
≥ 4 mm	0.6 (0–1.8)	0 (0–0)	1.2 (1.2–2.4)	1.9 (1.2–4.2)	12.3 (6.5–18.7)	< 0.001
≥ 5 mm	0 (0–0.6)	0 (0–0)	0.6 (0–1.2)	0.6 (0–1.3)	6.8 (3.6–10.7)	< 0.001
≥ 6 mm	0 (0–0)	0 (0–0)	0 (0–0)	0 (0–0)	2.5 (1.2–4.2)	< 0.001
**Percentage of teeth with PPD**^**b**^						
≥ 3 mm	28.6 (12.0–53.8)	17.9 (7.1–32.1)	39.6 (21.8–62.5)	46.4 (28.6–72.7)	78.6 (57.1–89.3)	< 0.001
≥ 4 mm	3.6 (0–7.7)	0 (0–0)	5.6 (3.6–10.7)	10.7 (7.1–16.7)	39.3 (23.1–52.4)	< 0.001
≥ 5 mm	0 (0–3.6)	0 (0–0)	3.6 (0–3.6)	3.6 (0–7.1)	23.1 (14.3–34.6)	< 0.001
≥ 6 mm	0 (0–0)	0 (0–0)	0 (0–0)	0 (0–0)	8.7 (7.1–15.4)	< 0.001
Average CAL (mm)^b^	1.8 (1.6–2.1)	1.7 (1.5–1.8)	1.9 (1.6–2.0)	2.0 (1.8–2.4)	2.7 (2.4–3.1)	< 0.001
**Percentage of sites with CAL**^**b**^						
≥ 3 mm	12.3 (5.4–23.2)	6.5 (3.1–12.5)	14.0 (9.5–20.9)	23.8 (14.9–37.0)	47.6 (32.7–60.3)	< 0.001
≥ 4 mm	0.7 (0–3.0)	0 (0–0.6)	1.2 (0.6–1.3)	3.6 (2.4–7.1)	19.6 (12.3–26.2)	< 0.001
≥ 5 mm	0 (0–0.6)	0 (0–0)	0.6 (0–1.2)	0.6 (0–1.8)	9.3 (4.8–13.9)	< 0.001
≥ 6 mm	0 (0–0)	0 (0–0)	0 (0–0)	0 (0–0.6)	3.1 (1.9–6.1)	< 0.001
**Percentage of teeth with CAL**^**b**^						
≥ 3 mm	37.5 (21.4–59.3)	25.0 (14.3–39.3)	42.9 (31.5–53.7)	57.1 (40.7–79.2)	82.6 (69.2–95.5)	< 0.001
≥ 4 mm	3.7 (0–13.6)	0 (0–3.6)	3.6 (3.6–7.1)	14.3 (9.1–26.9)	50.0 (33.3–68.0)	< 0.001
≥ 5 mm	0 (0–3.6)	0 (0–0)	3.6 (0–3.7)	3.6 (0–7.7)	25.0 (16.0–39.1)	< 0.001
≥ 6 mm	0 (0–0)	0 (0–0)	0 (0–0)	0 (0–3.6)	11.1 (7.4–20.0)	< 0.001
BOP (%)^b^	5.4 (1.8–13.1)	3.0 (0.6–9.5)	8.0 (3.0–19.0)	8.9 (3.0–17.3)	17.9 (8.9–29.2)	< 0.001
PISA (mm^2^)^b^	62 (16–164)	31 (6–97)	93 (37–205)	114 (37–251)	348 (196–561)	< 0.001
**Number of teeth lost**^**b**^	0 (0–1)	0 (0–1)	0 (0–1)	0 (0–2)	1 (0–4)	< 0.001
Number of teeth lost categories^c^						< 0.001
None	587 (61.9%)	353 (65.2%)	46 (71.9%)	162 (56.8%)	26 (44.1%)	
1–5	330 (34.8%)	180 (33.3%)	14 (21.9%)	111 (38.9%)	25 (42.4%)	
≥ 6	32 (3.4%)	8 (1.5%)	4 (6.3%)	12 (4.2%)	8 (13.6%)	
Denture use^c^	14 (1.5%)	2 (0.4%)	2 (3.1%)	6 (2.1%)	4 (6.8%)	< 0.001
OHI-S^b^	0.3 (0–0.8)	0.2 (0–0.6)	0.4 (0.2–0.9)	0.5 (0.2–1.0)	0.8 (0.3–1.3)	< 0.001
Tooth brushing ≥ 2 times/day^c^	890 (93.8%)	510 (94.3%)	62 (96.9%)	263 (92.3%)	55 (93.2%)	0.493
Regular dental check-ups^c^	421 (44.4%)	246 (45.5%)	29 (45.3%)	115 (40.4%)	31 (52.5%)	0.290
**Age**^**a**^	43.2 (12.2)	41.1 (11.3)	41.1 (11.8)	45.9 (12.7)	51.7 (11.7)	< 0.001
Age categories^c^						< 0.001
< 45 years	540 (56.9%)	350 (64.7%)	40 (62.5%)	134 (47.0%)	16 (27.1%)	
45–54 years	207 (21.8%)	110 (20.3%)	13 (20.3%)	68 (23.9%)	16 (27.1%)	
≥ 55 years	202 (21.3%)	81 (15.0%)	11 (17.2%)	83 (29.1%)	27 (45.8%)	
Sex (male)^c^	267 (28.1%)	111 (20.5%)	21 (32.8%)	108 (37.9%)	27 (45.8%)	< 0.001
Current smoker^c^	132 (13.9%)	52 (9.6%)	11 (17.2%)	52 (18.2%)	17 (28.8%)	< 0.001
Overweight^c^	189 (19.9%)	79 (14.6%)	13 (20.3%)	69 (24.2%)	28 (47.5%)	< 0.001
Diabetes mellitus^c^	65 (6.8%)	25 (4.6%)	2 (3.1%)	31 (10.9%)	7 (11.9%)	0.002

AAP, American Academy of Periodontology; BOP, bleeding on probing; CAL, clinical attachment loss; CDC, Centers for Disease Control and Prevention; IQR, interquartile range; OHI-S, simplified oral hygiene index; PISA, periodontal inflamed surface area; PPD, periodontal probing depth; SD, standard deviation.

^a^Presented as mean (SD).

^b^Presented as median (IQR).

^c^Presented as N (%).

### Responses to the 9 self-reported questions by periodontitis classification

Table 2 presents the frequency of responses to the 9 self-reported questions and the crude ORs for periodontitis outcomes. The frequency of “Don’t know” responses ranged from 4.4% for item 4, “Loose tooth” to 28.3% for item 1, “Have gum disease.” The frequency of “Refused” responses was low, with the highest proportion observed for item 7, “Floss use,” with 6.4% refusals. Responses to self-reported question items 1, 2, 4, 5, 6, and 9 were significantly associated with periodontitis, regardless of the severity category used. Items 1–6, excluding the “I don’t know” responses, did not significantly attenuate the association between responses to the self-reported questions and periodontitis outcomes (Table S3).

**Table 2:** Responses to questions stratified by periodontitis status among Japanese adults (N = 949).

Item	Question (abbreviation) and response (codes for logistic regression)	Total	Periodontitis severity based on CDC/AAP definition				p*	Crude odds ratio		
								(95% confidence interval)		
			No	Mild	Moderate	Severe		*p*^†^		
								Severe (1) vs. no/mild/moderate (0)	Moderate/severe (1) vs. no/mild (0)	Mild/moderate/severe (1) vs. no (0)
		N = 949	n = 541	n = 64	n = 285	n = 59				
1	Do you think you might have gum disease? (have gum disease)						< 0.001			
	Yes (1)	358 (37.7%)	165 (30.5%)	29 (45.3%)	123 (43.2%)	41 (69.5%)		4.10	1.95	2.06
	No (0)	319 (33.6%)	211 (39.0%)	19 (29.7%)	80 (28.1%)	9 (15.3%)		(2.31–7.25)	(1.48–2.56)	(1.58–2.69)
	Don’t know (0)	269 (28.3%)	164 (30.3%)	16 (25.0%)	80 (28.1%)	9 (15.3%)		< 0.001	< 0.001	< 0.001
	Refused	3 (0.3%)	1 (0.2%)	0 (0%)	2 (0.7%)	0 (0%)				
2	Overall, how would you rate the health of your teeth and gums? (teeth/gum health)						< 0.001	3.75	2.21	2.01
	Excellent (0)	1 (0.1%)	1 (0.2%)	0 (0%)	0 (0%)	0 (0%)		(2.15–6.55)	(1.68–2.90)	(1.53–2.62)
	Very good (0)	7 (0.7%)	5 (0.9%)	0 (0%)	1 (0.4%)	1 (1.7%)		< 0.001	< 0.001	< 0.001
	Good (0)	55 (5.8%)	36 (6.7%)	1 (1.6%)	17 (6.0%)	1 (1.7%)				
	Fair (0)	459 (48.4%)	287 (53.0%)	36 (56.3%)	119 (41.8%)	17 (28.8%)				
	Poor (1)	343 (36.1%)	158 (29.2%)	20 (31.3%)	126 (44.2%)	39 (66.1%)				
	Don’t know (0)	83 (8.7%)	53 (9.8%)	7 (10.9%)	22 (7.7%)	1 (1.7%)				
	Refused	1 (0.1%)	1 (0.2%)	0 (0%)	0 (0%)	0 (0%)				
3	Have you ever had treatment for gum disease such as scaling and root planing, sometimes called “deep cleaning”? (had gum treatment)						0.28	1.90	1.17	1.12
	Yes (1)	507 (53.4%)	283 (52.3%)	32 (50.0%)	152 (53.3%)	40 (67.8%)		(1.08–3.34)	(0.90–1.53)	(0.86–1.44)
	No (0)	329 (34.7%)	187 (34.6%)	27 (42.2%)	102 (35.8%)	13 (22.0%)		0.025	0.246	0.405
	Don’t know (0)	112 (11.8%)	71 (13.1%)	5 (7.8%)	30 (10.5%)	6 (10.2%)				
	Refused	1 (0.1%)	0 (0%)	0 (0%)	1 (0.4%)	0 (0%)				
4	Have you ever had any teeth become loose on their own, without an injury? (loose tooth)						< 0.001	8.81	2.58	2.79
	Yes (1)	117 (12.3%)	41 (7.6%)	10 (15.6%)	37 (13.0%)	29 (49.2%)		(5.05–15.36)	(1.74–3.82)	(1.86–4.18)
	No (0)	790 (83.2%)	478 (88.4%)	50 (78.1%)	236 (82.8%)	26 (44.1%)		< 0.001	< 0.001	< 0.001
	Don’t know (0)	42 (4.4%)	22 (4.1%)	4 (6.3%)	12 (4.2%)	4 (6.8%)				
	Refused	0 (0%)	0 (0%)	0 (0%)	0 (0%)	0 (0%)				
5	Have you ever been told by a dental professional that you lost bone around your teeth? (lost bone)						< 0.001	8.09	3.11	2.95
	Yes (1)	73 (7.7%)	24 (4.4%)	4 (6.3%)	25 (8.8%)	20 (33.9%)		(4.41–14.83)	(1.90–5.09)	(1.78–4.89)
	No (0)	812 (85.6%)	488 (90.2%)	55 (85.9%)	236 (82.8%)	33 (55.9%)		< 0.001	< 0.001	< 0.001
	Don’t know (0)	63 (6.6%)	29 (5.4%)	5 (7.8%)	23 (8.1%)	6 (10.2%)				
	Refused	1 (0.1%)	0 (0%)	0 (0%)	1 (0.4%)	0 (0%)				
6	During the past three months, have you noticed a tooth that doesn’t look right? (tooth does not look right)						0.006	2.35	1.73	1.93
	Yes (1)	148 (15.6%)	64 (11.8%)	14 (21.9%)	53 (18.6%)	17 (28.8%)		(1.30–4.24)	(1.21–2.46)	(1.36–2.75)
	No (0)	692 (72.9%)	416 (76.9%)	42 (65.6%)	198 (69.5%)	36 (61.0%)		0.005	0.003	< 0.001
	Don’t know (0)	109 (11.5%)	61 (11.3%)	8 (12.5%)	34 (11.9%)	6 (10.2%)				
	Refused	0 (0%)	0 (0%)	0 (0%)	0 (0%)	0 (0%)				
7	Aside from brushing your teeth with a toothbrush, in the last 7 days, how many days did you use dental floss or any other device to clean between your teeth? (floss use)						0.13	0.90	1.05	1.19
	1–7 days (0)	474 (49.9%)	283 (52.3%)	26 (40.6%)	136 (47.7%)	29 (49.2%)		(0.51–1.59)	(0.80–1.39)	(0.91–1.55)
	Never (1)	414 (43.6%)	230 (42.5%)	35 (54.7%)	126 (44.2%)	23 (39.0%)		0.722	0.714	0.212
	Refused	61 (6.4%)	28 (5.2%)	3 (4.7%)	23 (8.1%)	7 (11.9%)				
8	Aside from brushing your teeth with a toothbrush, in the last 7 days, how many times did you use mouthwash or other dental rinse product that you use to treat dental disease or dental problems? (mouthwash)						0.17	0.79	1.02	1.07
	Never (0)	646 (68.1%)	377 (69.7%)	42 (65.6%)	186 (65.3%)	41 (69.5%)		(0.42–1.50)	(0.75–1.38)	(0.80–1.44)
	1–7 days (1)	256 (27.0%)	145 (26.8%)	20 (31.3%)	78 (27.4%)	13 (22.0%)		0.47	0.908	0.637
	Refused	47 (5.0%)	19 (3.5%)	2 (3.1%)	21 (7.4%)	5 (8.5%)				
9	During the past three months, have you had bleeding gums? (Bleeding gums)						< 0.001	3.50	1.66	1.63
	Never (0)	191 (20.1%)	124 (22.9%)	7 (10.9%)	57 (20.0%)	3 (5.1%)		(1.96–6.25)	(1.27–2.17)	(1.25–2.11)
	Hardly ever (0)	348 (36.7%)	211 (39.0%)	29 (45.3%)	94 (33.0%)	14 (23.7%)		< 0.001	< 0.001	< 0.001
	Sometimes (1)	316 (33.3%)	157 (29.0%)	23 (35.9%)	103 (36.1%)	33 (55.9%)				
	Fairly often (1)	79 (8.3%)	44 (8.1%)	3 (4.7%)	25 (8.8%)	7 (11.9%)				
	Very often (1)	15 (1.6%)	5 (0.9%)	2 (3.1%)	6 (2.1%)	2 (3.4%)				

AAP, American Academy of Periodontology; CDC, Centers for Disease Control and Prevention.

*p-value for the chi-squared test for the association between periodontal classification and responses to questions.

^†^*p*-value for the logistic regression analyses for the association between periodontal classification and responses to questions.

Table 3 presents the correlation results between responses to any pair of self-reported questions. Significant moderate correlations were found between “Have gum disease” and “Teeth/gum health” (*p* < 0.05; Kendall’s Tau-b correlation coefficient = 0.565) and between “Loose tooth” and “Lost bone” (*p* < 0.05; Kendall’s Tau-b correlation coefficient = 0.440). All the other bivariate correlations were weak (Kendall’s Tau-b correlation coefficient < 0.4) or non-significant.

**Table 3:** Correlations between pair-wise dichotomized periodontitis questionnaire items.

Items		1	2	3	4	5	6	7	8
1	Have gum disease (1 = yes, 0 = no)								
2	Teeth/gum health (1 = poor, 0 = fair/good/very good/excellent)	0.565*							
3	Had gum treatment (1 = yes, 0 = no)	0.007	− 0.011						
4	Loose tooth (1 = yes, 0 = no)	0.279*	0.320*	0.105*					
5	Lost bone (1 = yes, 0 = no)	0.199*	0.197*	0.191*	0.440*				
6	Tooth does not look right (1 = yes, 0 = no)	0.207*	0.248*	− 0.014	0.229*	0.163*			
7	Floss use (1 = never, 0 = 1 to 7 days)	− 0.106*	0.006	− 0.146*	− 0.025	− 0.104*	0.029		
8	Mouthwash (1 = 1 to 7 days, 0 = never)	0.105*	0.082*	0.067*	0.108*	0.015	0.051	− 0.269*	
9	Bleeding gums (1 = yes, 0 = no)	0.203*	0.211*	− 0.034	0.069*	0.004	0.135*	0.082*	0.050

Kendall’s Tau-b correlation coefficients are presented.

*p < 0.05.

### Predictive ability of the self-reported questions

Table 4 presents the results of the multivariable logistic regression analyses, which included participants without “refused” responses to any questions (*n* = 883). Adding the questions on bleeding gums contributed to a significant improvement in predicting severe periodontitis (AUC = 0.79 in Model 1 and 0.85 in Model 2; *p* = 0.005). The best reduced model for the self-reported oral health questions (Model 5) that predicted severe periodontitis and total periodontitis (i.e., combined mild, moderate, and severe periodontitis) included the same oral health questions, namely: “Have gum disease,” “Loose tooth,” “Lost bone,” and “Bleeding gums.” Model 5 had an AUC of 0.83 with a sensitivity of 73.1% and specificity of 74.3% for predicting severe periodontitis. The combined use of self-reported questions and demographic and health-related variables performed well in predicting periodontitis. The parsimonious model (Model 6) for predicting severe periodontitis had an AUC of 0.87 with a sensitivity of 75.0% and specificity of 81.5%. Model 6, used to predict the combined severe and moderate periodontitis, had an AUC of 0.72 with a sensitivity of 70.8% and specificity of 60.1%. Model 6, used to predict total periodontitis, had an AUC of 0.71 with a sensitivity of 72.1% and specificity of 59.8%. After testing all possible combinations, the best significant subsets of oral health questions were selected in the following parsimonious models: for severe periodontitis, four oral health questions (“Have gum disease,” “Loose tooth,” “Lost bone,” and “Bleeding gums”); for combined severe and moderate periodontitis, three oral health questions (“Have gum disease,” “Teeth/gum health,” and “Bleeding gums”); and for total periodontitis, two oral health questions (“Have gum disease” and “Bleeding gums”).

**Table 4:** Logistic regression models for periodontitis among Japanese adults (N = 883).

	Outcomes																	
	CDC/AAP case definition																	
	Severe periodontitis						Moderate/severe periodontitis						Mild/moderate/severe periodontitis					
	Prevalence = 5.9%						Prevalence = 35.3%						Prevalence = 42.2%					
	Model 1	Model 2	Model 3	Model 4	Model 5	Model 6	Model 1	Model 2	Model 3	Model 4	Model 5	Model 6	Model 1	Model 2	Model 3	Model 4	Model 5	Model 6
**Predictor variables**																		
Nine-item self-reported questionnaire																		
1. Have gum disease	X	X		X	X	X	X	X		X		X	X	X		X	X	X
2. Teeth/gum health	X	X		X			X	X		X	X	X	X	X		X		
3. Had gum treatment	X	X		X			X	X		X			X	X		X		
4. Loose tooth	X	X		X	X	X	X	X		X	X		X	X		X	X	
5. Lost bone	X	X		X	X	X	X	X		X	X		X	X		X	X	
6. Tooth does not look right	X	X		X			X	X		X			X	X		X		
7. Floss use	X	X		X			X	X		X			X	X		X		
8. Mouthwash	X	X		X			X	X		X			X	X		X		
9. Bleeding gums		X		X	X	X		X		X	X	X		X		X	X	X
**Demographic and health-related variables**																		
Age			X	X		X			X	X		X			X	X		X
Sex			X	X		X			X	X		X			X	X		X
Current smoking			X	X					X	X		X			X	X		X
Diabetes mellitus			X	X					X	X		X			X	X		
Overweight			X	X		X			X	X		X			X	X		X
Tooth loss			X	X					X	X					X	X		
AUC	0.79	0.85	0.78	0.88	0.83	0.87	0.63	0.64	0.69	0.72	0.63	0.72	0.63	0.64	0.69	0.72	0.63	0.71
Sensitivity	65.4	78.9	67.3	80.8	73.1	75.0	47.8	53.2	57.1	62.5	52.9	70.8	57.6	47.5	69.2	75.9	71.3	72.1
Specificity	82.6	73.9	75.7	77.1	74.3	81.5	73.2	64.8	68.8	69.4	66.9	60.1	63.1	72.0	60.4	56.5	44.3	59.8
BIC	380	372	402	396	344	344	1158	1162	1110	1136	1132	1088	1210	1214	1169	1191	1186	1140

Model 1 includes the 8-item self-reported questionnaire for oral health.

Model 2 added the question on bleeding gums to Model 1.

Model 3 includes demographic and health-related variables.

Model 4 includes a combination of self-reported oral health questions and demographic and health-related variables (full model).

Model 5 includes the best significant subset of self-reported oral health questions (the parsimonious model, for the self-reported oral health questions).

Model 6 includes the best significant subset selected from the full model.

AAP, American Academy of Periodontology; AUC, area under the receiver operating characteristic curve; BIC, Bayesian Information Criteria; CDC, Centers for Disease Control and Prevention.

Sensitivity analyses showed that the combined use of self-reported questions and demographic and health-related variables performed well in predicting periodontitis based on other definitions of periodontitis categories. Model 6 had an AUC of 0.76, sensitivity of 69.9%, and specificity of 68.3% for predicting EFP/AAP severe periodontitis; and an AUC of 0.71, sensitivity of 61.9%, and specificity of 64.5% for predicting the highest quintile of PISA.

### Development of the screening score for severe periodontitis based on the self-reported oral health questions

As presented in the previous paragraphs, our models including the self-reported questions had good predictive ability for periodontitis, especially the severe cases. Table 5 presents the screening score with and without weighting that can be used for predicting the CDC/AAP severe periodontitis. The weighted score was calculated using the coefficients obtained based on Model 5 for the CDC/AAP severe periodontitis in Table 4. The weighted screening score ranged from 0 to 47, whereas the unweighted screening score ranged from 0 to 4. The two receiver operating characteristic curves were plotted, and their areas compared (Figure S1). Using the weighted screening score, this showed a small contribution to improving the predictive ability (AUC = 0.82 for unweighted score; AUC = 0.83 for weighted score). The difference in AUCs did not reach statistical significance (*p* = 0.07). The cut-off of the unweighted score to best differentiate between individuals with and without severe periodontitis was 2, with a sensitivity of 73.1% and specificity of 74.3%.

**Table 5:** Multivariable regression coefficients and screening score for periodontitis.

Outcome = CDC/AAP severe periodontitis			
Predictor variables	Coefficient	Score with weighting^a^	Score without weighting
Have gum disease (1 = yes, 0 = no)	0.70	7	1
Loose tooth (1 = yes, 0 = no)	1.63	16	1
Lost bone (1 = yes, 0 = no)	1.21	12	1
Bleeding gums (1 = yes, 0 = no)	1.21	12	1
	Score range	0–47	0–4
	Cut-off	19	2
	AUC	0.83	0.82
	Sensitivity	73.1%	73.1%
	Specificity	74.3%	74.3%

AAP, American Academy of Periodontology; AUC, area under the receiver operating characteristic curve; CDC, Centers for Disease Control and Prevention.

^a^The weighted score was calculated using the coefficients obtained from the parsimonious model for the self-reported oral health questions.

## Discussion

In this study, we developed a 9-item self-reported questionnaire for periodontitis by translating the 8 items included in the CDC/AAP questionnaire and one item on gingival bleeding. The translation of the questionnaire for use in the Japanese context was performed using a rigorous methodological approach. The cognitive test revealed that the questionnaire had good language clarity. The expert panel found the questionnaire to be an equivalent measure of the original English version with a good content-related validity.

Next, we evaluated the performance of the self-reported questionnaire in predicting periodontitis according to different definitions. The parsimonious multivariable models, including the self-reported oral health questions and socio-demographic and health-related variables (Model 6 in Tables 4 and S4) had an AUC of > 0.70 (range, 0.71–0.87) for any combination of periodontitis case-definitions, which was considered useful or moderate predictive performance^32^. The diagnosis of periodontitis at the population level remains challenging. Periodontitis surveillance at the workplace or the community level is rarely performed in Japan^25,33^. The present study results demonstrated that self-report questionnaires can be an alternative tool with acceptable validity and reliability to screen individuals with periodontitis. In addition, the self-reported questionnaire is a less resource-demanding approach than clinical periodontal examinations.

In our study, multivariable prediction models including self-reported oral health questions with demographic characteristics and risk factors performed well in predicting severe cases of periodontitis (AUC = 0.87). Our results are comparable to those of other studies. Multivariable prediction models for severe periodontitis had an AUC of 0.83 in the NHANES participants (American population)^12^, 0.85 in the Black Women’s Health Study participants (U.S. population)^19^, 0.82 in a French population^20^, 0.75 in the Di@bet.es Study participants (Spanish population)^21^, 0.82 in a Dutch population^22^, and 0.81 in a Korean population^23^.

According to a recent systematic review^34^, the sensitivity and specificity of a self-report questionnaire ranged from 4 to 93% and 58 to 94%, respectively. This high heterogeneity in predictive performance may be due to multiple causes, including variations in periodontitis case definitions, items included in the questionnaire, and study population characteristics. Among these, the periodontitis case definition is one of the major reasons for the study results’ heterogeneity. In our study, the predictive ability varied with the definition used for periodontitis. Generally, periodontitis progresses silently, and the pathological changes take a long time before pain, discomfort, and functional disability occur^35^. This poor self-perception of periodontitis progression can partly explain the lower predictive ability of the self-report in milder cases of periodontitis in our study.

We demonstrated that the best reduced model including only the oral health questions performed at an acceptable statistical threshold for severe periodontitis (AUC = 0.83). It would be more useful for the screening score to exclude non-oral variables when the score is used in epidemiological studies investigating the link between periodontitis and systemic conditions. If the score had included non-oral variables, such as age and sex, comparison of the screening score according to age and sex could not be performed. In addition, the statistical model could not simultaneously include the score, age, and sex due to the multicollinearity among those variables. Therefore, we proposed the screening score based on oral health questions only. The screening score is a user-friendly measure and is a potentially useful tool for future epidemiological studies.

We demonstrated that adding the question on gum bleeding improved the predictive performance of the questionnaire. Additionally, the best reduced model of oral health variables for every periodontitis case definition used in this study included the question on gum bleeding. Gingival bleeding is an indicator of the presence of an inflammatory lesion. Gingival bleeding is objective^35^, and therefore, may be less confusing than other items, such as teeth/gum health, for our study participants. One study^25^ demonstrated the usefulness of using responses to both oral health questions and smoking status to screen for severe periodontitis in Japanese workers. As seen in our study result, the question about gum bleeding was selected for the prediction model in that study. Although the findings were novel, their study defined periodontitis based on PMPE, which cannot be considered a gold standard diagnosis. Moreover, the study population consisted of only male workers, and their age range was very narrow (i.e., between 50 and 59 years).

This study has several strengths. First, we included a sufficient number of participants for the evaluation of validity of the self-reported measures to predict periodontitis. The sample size was estimated based on the prevalence of severe periodontitis observed in a previous study conducted at a workplace in Japan^27^. To the best of our knowledge, this is the largest study conducted to assess the validity of the CDC/AAP questionnaire in a non-American population. Second, we performed FMPE, which is considered the gold standard for periodontitis diagnosis in population studies^12^. Third, the diagnostic accuracy of self-reported measures was tested against multiple case definitions, including the CDC/AAP definition for periodontitis^30^. The CDC/AAP definition is specifically designed for population-based surveillance^30^, which met the purpose of our study of assessing the validity of a self-report questionnaire for population-based surveillance. Furthermore, the CDC/AAP definition is the most frequently used and is considered the most appropriate definition in an epidemiological setting^36^.

This study also has some limitations. First, selection bias might have occurred as the data collection was performed on a voluntary basis. Of 1993 individuals who were invited to our study, 1045 agreed to participate. There were no significant differences in age and sex between individuals who agreed and declined to participate (Table S5). However, we could not investigate whether there were differences in other characteristics, such as periodontal and medical conditions, between these two groups. The national survey in Japan, the SDD, does not have the FMPE data. When the periodontitis definition used in the SDD 2016 was applied to the current study population, the prevalence of periodontitis defined as pocket scores of 1 or 2 of the modified Community Periodontal Index, was 55.6%. Although no large difference was observed in the prevalence of periodontitis between this study and the SDD 2016 (i.e., 49.4%), future studies are needed to verify whether current findings can be applied to the broader population. Second, although we showed that the locally adapted version of the self-report questionnaire had acceptable validity for predicting periodontitis in this study population, the test–retest reliability for the questionnaire could not tested because no self-report questionnaires were repeated for participants.

In conclusion, we performed the translation of and local modification to existing English-version questions and developed a Japanese 9-item self-report questionnaire on periodontitis. Then, we assessed the validity of the self-report questionnaire in a cohort of Japanese adults. Finally, we generated four oral health question-based screening scores based on the model described, including the best significant subset of self-reported oral health questions. Our study demonstrated the following: (1) a self-report questionnaire has acceptable diagnostic capacity for the detection of periodontitis in a Japanese population and is potentially useful for population-based surveillance; and (2) the screening score is a user-friendly tool that can be a viable alternative to determine severe periodontitis in epidemiologic studies when the clinical periodontal examination is impractical, due to time and/or resource constraints.

## Supplementary Information

Supplementary Information 1.

Supplementary Information 2.

## Data Availability

The data presented in this study are available on request from the corresponding author. The data are not publicly available due to ethical and legal restrictions imposed by the Ethics Committee of Kyushu Dental University.
