# Author Correction: Validation of a self-report questionnaire for periodontitis in a Japanese population

**DOI:** 10.1038/s41598-021-97275-7

**Published:** 2021-08-30

**Authors:** Masanori Iwasaki, Michihiko Usui, Wataru Ariyoshi, Keisuke Nakashima, Yoshie Nagai‑Yoshioka, Maki Inoue, Kaoru Kobayashi, Wenche S. Borgnakke, George W. Taylor, Tatsuji Nishihara

**Affiliations:** 1 Tokyo Metropolitan Institute of Gerontology, 35‑2 Sakae‑cho, Itabashi‑Ku, Tokyo, 173‑0015 Japan; 2 Division of Periodontology, Kyushu Dental University, Kitakyushu, Japan; 3 Division of Infections and Molecular Biology, Kyushu Dental University, Kitakyushu, Japan; 4 Dental Center for Regional Medical Survey, Kyushu Dental University, Kitakyushu, Japan; 5 Graduate School of Dentistry, MSc Program, Kyushu Dental University, Kitakyushu, Japan; 6 Department of Periodontics and Oral Medicine, University of Michigan School of Dentistry, Ann Arbor, MI USA; 7 Division of Oral Epidemiology and Dental Public Health, Department of Preventive and Restorative Dental Sciences, UCSF School of Dentistry, San Francisco, CA USA

Correction to: *Scientific Reports* 10.1038/s41598-021-93965-4, published online 23 July 2021

In the original version of this Article, the Supplementary Information 1 file contained tracked changes.

In addition, the titles and heading rows for Tables S1, S2, S3 and S4 were incomplete.

Lastly, the columns in Table S5 have been rearranged for improved readability.

The original Supplementary Information 1 file is provided below.

These errors have now been corrected in the Supplementary Information 1 file that accompanies the original Article.

## Supplementary Information

Supplementary Information 1.

