# Supplementary material for: Validation of a self-report questionnaire for periodontitis in a Japanese population
Source: Sci Rep. 2021 Jul 23;11:15078. doi: 10.1038/s41598-021-93965-4 (PMC8302714; doi:10.1038/s41598-021-93965-4)
Supplement: Supplementary file 1 — Supplementary Information 1. [file 41598_2021_93965_MOESM1_ESM.docx]

**SUPPLEMENTARY MATERIAL**

**Validation of a Self-report Questionnaire for Periodontitis**

**in a Japanese Population**

Masanori Iwasaki^1*^, Michihiko Usui^2^, Wataru Ariyoshi^3^, Keisuke Nakashima^2^, Yoshie Nagai-Yoshioka^3^, Maki Inoue^4^, Kaoru Kobayashi^5^, Wenche S. Borgnakke^6^, George W. Taylor^7^, Tatsuji Nishihara^4^

^1^ Tokyo Metropolitan Institute of Gerontology, Tokyo, Japan

^2^ Division of Periodontology, Kyushu Dental University, Kitakyushu, Japan

^3^ Division of Infections and Molecular Biology, Kyushu Dental University, Kitakyushu, Japan

^4^ Dental Center for Regional Medical Survey, Kyushu Dental University, Kitakyushu, Japan

^5^ Graduate School of Dentistry, MSc Program, Kyushu Dental University, Kitakyushu, Japan

^6^ Department of Periodontics and Oral Medicine, University of Michigan School of Dentistry, Ann Arbor, MI, USA

^7^ Department of Preventive and Restorative Dental Sciences, Division of Oral Epidemiology and Dental Public Health, UCSF School of Dentistry, CA, USA

**Corresponding Author:** Masanori Iwasaki

Tokyo Metropolitan Institute of Gerontology

35-2 Sakae-cho, Itabashi-Ku, Tokyo 173-0015, Japan

Tel +81 33 964 3241 Ext 4215

Fax +81 33 964 2316

E-mail: iwasaki@tmig.or.jp

| **Table S1.** Self-reported oral health questions in English and in Japanese. | | |
| --- | --- | --- |
| Item | Original Questions and Responses in the English Version 1 (EV1) | Final Questions and Responses in the Japanese Version 4 (JV4) |
| 1 | Do you think you might have gum disease? | 歯ぐきの病気にかかっているかもしれないと思いますか？ |
|  | (Yes, No, Refused, Don’t Know) | (はい, いいえ, 答えたくない, わからない) |
| 2 | Overall, how would you rate the health of your teeth and gums? | 全体として、歯と歯ぐきの健康状態についてどう思いますか？ |
|  | (Excellent, Very good, Good, Fair, Poor, Refused, Don’t Know) | (すばらしい, とてもよい, よい, ふつう, わるい, 答えたくない, わからない) |
| 3 | Have you ever had treatment for gum disease such as scaling and root planing, sometimes called “deep cleaning”? | スケーリング&ルートプレーニングなどの歯ぐきの病気の治療を受けたことがありますか？ ※「スケーリング&ルートプレーニング」とは「歯に付着している歯垢や歯石を専用の器具を使って取り除き、歯の根の表面をなめらかにすること」です。 |
|  | (Yes, No, Refused, Don’t Know) | (はい, いいえ, 答えたくない, わからない) |
| 4 | Have you ever had any teeth become loose on their own, without an injury? | 自然と歯がぐらつくようになったことはありますか？（怪我によるものは除きます。） |
|  | (Yes, No, Refused, Don’t Know) | (はい, いいえ, 答えたくない, わからない) |
| 5 | Have you ever been told by a dental professional that you lost bone around your teeth? | 歯医者あるいは歯科衛生士から「歯のまわりの骨が失われている」と言われたことがありますか？ |
|  | (Yes, No, Refused, Don’t Know) | (はい, いいえ, 答えたくない, わからない) |
| 6 | During the past three months, have you noticed a tooth that doesn’t look right? | 過去3ヶ月間に、見た目が正常ではない歯に気づいたことはありますか？ |
|  | (Yes, No, Refused, Don’t Know) | (はい, いいえ, 答えたくない, わからない) |
| 7 | Aside from brushing your teeth with a toothbrush, in the last seven days, how many times did you use dental floss or any other device to clean between your teeth? | 歯ブラシによる歯みがき以外で、過去7日間に、デンタルフロスなどを使った歯と歯の間の清掃を何日行いましたか？ |
|  | (___: Number of days, 77 = Refused) | （ []日, 答えたくない) |
| 8 | Aside from brushing your teeth with a toothbrush, in the last seven days, how many times did you use mouthwash or other dental rinse product that you use to treat dental disease or dental problems? | 歯ブラシによる歯みがき以外で、過去7日間に、口の中の病気やトラブルの治療のために うがい薬・洗口剤を何日使用しましたか？ |
|  | (___: Number of days, 77 = Refused) | （ []日, 答えたくない) |
| 9 | During the past three months, have you had bleeding gums? | ここ3ヵ月間で、歯ぐきから血が出たことはありますか？ |
|  | (Never, Hardly ever, Sometimes, Fairly often, Very often) | (まったくない, ほとんどない, 時々, しばしば, いつも) |

| **Table S2.** Cognitive test results of the Japanese questionnaire (N=100) | | | | | |
| --- | --- | --- | --- | --- | --- |
| Item | Responses to the questions | n |  | Level of agreement on the clarity of each question  (Rated on 5-point Likert scale) | n |
| 1 | Do you think you might have gum disease? |  |  |  |  |
|  | Yes | 36 |  | Strongly agree | 36 |
|  | No | 48 |  | Agree | 37 |
|  | Don’t Know | 16 |  | Neutral | 19 |
|  | Refused | 0 |  | Disagree | 5 |
|  |  |  |  | Strongly disagree | 3 |
| 2 | Overall, how would you rate the health of your teeth and gums? |  |  |  |  |
|  | Excellent | 2 |  | Strongly agree | 29 |
|  | Very good | 5 |  | Agree | 45 |
|  | Good | 16 |  | Neutral | 22 |
|  | Fair | 39 |  | Disagree | 3 |
|  | Poor | 33 |  | Strongly disagree | 1 |
|  | Refused | 0 |  |  |  |
|  | Don’t Know | 5 |  |  |  |
| 3 | Have you ever had treatment for gum disease such as scaling and root planing, sometimes called “deep cleaning”? |  |  |  |  |
|  | Yes | 9 |  | Strongly agree | 36 |
|  | No | 74 |  | Agree | 16 |
|  | Don’t Know | 16 |  | Neutral | 16 |
|  | Refused | 1 |  | Disagree | 15 |
|  |  |  |  | Strongly disagree | 17 |
| 4 | Have you ever had any teeth become loose on their own, without an injury? |  |  |  |  |
|  | Yes | 22 |  | Strongly agree | 62 |
|  | No | 73 |  | Agree | 29 |
|  | Don’t Know | 2 |  | Neutral | 7 |
|  | Refused | 3 |  | Disagree | 1 |
|  |  |  |  | Strongly disagree | 1 |
| 5 | Have you ever been told by a dental professional that you lost bone around your teeth? |  |  |  |  |
|  | Yes | 11 |  | Strongly agree | 63 |
|  | No | 87 |  | Agree | 27 |
|  | Don’t Know | 1 |  | Neutral | 6 |
|  | Refused | 1 |  | Disagree | 3 |
|  |  |  |  | Strongly disagree | 1 |
| 6 | During the past three months, have you noticed a tooth that doesn’t look right? |  |  |  |  |
|  | Yes | 15 |  | Strongly agree | 68 |
|  | No | 80 |  | Agree | 25 |
|  | Don’t Know | 4 |  | Neutral | 4 |
|  | Refused | 1 |  | Disagree | 2 |
|  |  |  |  | Strongly disagree | 1 |
| 7 | Aside from brushing your teeth with a toothbrush, in the last seven days, how many days did you use dental floss or any other device to clean between your teeth? |  |  |  |  |
|  | 1–7 days | 25 |  | Strongly agree | 70 |
|  | Never | 67 |  | Agree | 22 |
|  | Refused | 8 |  | Neutral | 4 |
|  |  |  |  | Disagree | 1 |
|  |  |  |  | Strongly disagree | 3 |
| 8 | Aside from brushing your teeth with a toothbrush, in the last seven days, how many times did you use mouthwash or other dental rinse product that you use to treat dental disease or dental problems? |  |  |  |  |
|  | Never | 67 |  | Strongly agree | 74 |
|  | 1–7 days | 27 |  | Agree | 20 |
|  | Refused | 6 |  | Neutral | 4 |
|  |  |  |  | Disagree | 0 |
|  |  |  |  | Strongly disagree | 2 |
| 9 | During the past three months, have you had bleeding gums? |  |  |  |  |
|  | Never | 29 |  | Strongly agree | 66 |
|  | Hardly ever | 34 |  | Agree | 27 |
|  | Sometimes | 30 |  | Neutral | 5 |
|  | Fairly often | 5 |  | Disagree | 1 |
|  | Very often | 2 |  | Strongly disagree | 1 |

| **Table S3.** Association between responses to questions* and clinically assessed periodontitis^30^ outcomes among Japanese adults (N=949) | | | | |
| --- | --- | --- | --- | --- |
|  | | Periodontitis categories compared | | |
| Item | Question (abbreviation) and response (codes for logistic regression) | Severe (1) *vs.* No/Mild/Moderate (0)  Crude Odds Ratio  (95% CI)  *p* | Moderate/Severe (1) *vs*. No/Mild (0)  Crude Odds Ratio  (95% CI)  *p* | Mild/Moderate/Severe (1) *vs.* No (0)  Crude Odds Ratio  (95% CI)  *p* |
| 1 | Do you think you might have gum disease? (Have gum disease) |  |  |  |
|  | Yes (1) | 4.45 | 2.18 | 2.29 |
|  | No (0) | (2.13–9.32) | (1.58–3.01) | (1.67–3.12) |
|  |  | <0.001 | <0.001 | <0.001 |
| 2 | Overall, how would you rate the health of your teeth and gums? (Teeth/gum health) | 3.40 | 2.17 | 2.00 |
|  | Excellent (0) | (1.93–5.98) | (1.64–2.89) | (1.51–2.63) |
|  | Very good (0) | <0.001 | <0.001 | <0.001 |
|  | Good (0) |  |  |  |
|  | Fair (0) |  |  |  |
|  | Poor (1) |  |  |  |
| 3 | Have you ever had treatment for gum disease such as scaling and root planing, sometimes called “deep cleaning”? (Had gum treatment) | 2.08 | 1.13 | 1.04 |
|  | Yes (1) | (1.10–3.96) | (0.85–1.51) | (0.79–1.38) |
|  | No (0) | 0.025 | 0.393 | 0.771 |
| 4 | Have you ever had any teeth become loose on their own, without an injury? (Loose tooth) | 9.68 | 2.61 | 2.84 |
|  | Yes (1) | (5.46–17.18) | (1.76–3.87) | (1.89–4.26) |
|  | No (0) | <0.001 | <0.001 | <0.001 |
|  | Don’t Know |  |  |  |
|  | Refused |  |  |  |
| 5 | Have you ever been told by a dental professional that you lost bone around your teeth? (Lost bone) | 8.91 | 3.24 | 3.08 |
|  | Yes (1) | (4.79–16.58) | (1.98–5.32) | (1.85–5.11) |
|  | No (0) | <0.001 | <0.001 | <0.001 |
| 6 | During the past three months, have you noticed a tooth that doesn’t look right? (Tooth does not look right) | 2.36 | 1.76 | 1.98 |
|  | Yes (1) | (1.29–4.34) | (1.23–2.52) | (1.38–2.83) |
|  | No (0) | 0.005 | 0.002 | <0.001 |

*Excluding “Don’t know” and “Refused” responses that were not assigned any 0 or 1 value for logistic regression analyses.

| **Table S4.** Logistic regression models for most severe periodontitis outcomes applying definitions other than the CDC/AAP^30^ among Japanese adults (N=883). | | | | | | | | | | | | |
| --- | --- | --- | --- | --- | --- | --- | --- | --- | --- | --- | --- | --- |
|  | Outcomes | | | | | | | | | | | |
|  | Severe periodontitis (EFP/AAP case definition^31^) | | | | | | Highest quintile of PISA^28^ | | | | | |
|  | Prevalence=15.4% | | | | | | Prevalence=19.9% | | | | | |
|  | Model 1 | Model 2 | Model 3 | Model 4 | Model 5 | Model 6 | Model 1 | Model 2 | Model 3 | Model 4 | Model 5 | Model 6 |
| Predictor variables |  |  |  |  |  |  |  |  |  |  |  |  |
| Nine-item self-reported questionnaire | | | | | | |  |  |  |  |  |  |
| 1. Have gum disease | X | X |  | X |  |  | X | X |  | X | X | X |
| 2. Teeth/gum health | X | X |  | X | X | X | X | X |  | X |  |  |
| 3. Had gum treatment | X | X |  | X |  |  | X | X |  | X |  |  |
| 4. Loose tooth | X | X |  | X | X | X | X | X |  | X |  |  |
| 5. Lost bone | X | X |  | X | X | X | X | X |  | X | X | X |
| 6. Tooth does not look right | X | X |  | X |  |  | X | X |  | X |  |  |
| 7. Floss use | X | X |  | X |  |  | X | X |  | X | X | X |
| 8. Mouthwash | X | X |  | X |  |  | X | X |  | X |  |  |
| 9. Bleeding gum |  | X |  | X | X | X |  | X |  | X | X | X |
| Demographic and health-related variables | | | | | | |  |  |  |  |  |  |
| Age |  |  | X | X |  | X |  |  | X | X |  |  |
| Sex |  |  | X | X |  | X |  |  | X | X |  | X |
| Current smoking |  |  | X | X |  |  |  |  | X | X |  |  |
| Diabetes mellitus |  |  | X | X |  |  |  |  | X | X |  |  |
| Overweight |  |  | X | X |  | X |  |  | X | X |  | X |
| Tooth loss |  |  | X | X |  |  |  |  | X | X |  |  |
| AUC | 0.69 | 0.72 | 0.70 | 0.77 | 0.72 | 0.76 | 0.64 | 0.66 | 0.65 | 0.71 | 0.66 | 0.71 |
| Sensitivity | 57.4 | 58.8 | 63.2 | 67.7 | 56.6 | 69.9 | 76.7 | 56.8 | 60.2 | 65.9 | 60.8 | 61.9 |
| Specificity | 72.7 | 74.0 | 68.4 | 75.1 | 75.4 | 68.3 | 42.7 | 67.5 | 65.4 | 65.2 | 61.8 | 64.5 |
| BIC | 734 | 731 | 752 | 748 | 702 | 695 | 907 | 902 | 899 | 917 | 872 | 853 |
| Model 1 includes the 8-item self-reported questionnaire for oral health. | | | | | | | | | | | | |
| Model 2 added the question on bleeding gums to Model 1. | | | | | | | | | | | | |
| Model 3 includes demographic and health-related variables. | | | | | | | | | | | | |
| Model 4 includes a combination of the self-reported oral health questions and demographic and health-related variables (full model). | | | | | | | | | | | | |
| Model 5 includes the best significant subset of self-reported oral health questions (the parsimonious model, for the self-reported oral health questions). | | | | | | | | | | | | |
| Model 6 includes the best significant subset of items selected from the full model. | | | | | | | | | | | | |
| AAP, American Academy of Periodontology; AUC, area under the receiver operating characteristic curve; BIC, Bayesian Information Criteria; EFP, European Federation of Periodontology; PISA, periodontal inflamed surface area | | | | | | | | | | | | |

| **Table S5.** Comparisons of individuals who declined or agreed to participate in the study by age and sex. | | | | |
| --- | --- | --- | --- | --- |
| **Characteristic** | **Declined** | **Agreed** | **Total** | ***p*** |
| Number (n) | 948 | 1,045 | 1,993 |  |
| Age, mean (SD), years | 43.5 (13.9) | 43.3 (12.2) | 43.4 (13.1) | 0.721 |
| Sex (Men), n (%) | 248 (26.2%) | 283 (27.1%) | 531 (26.6%) | 0.642 |
| SD, standard deviation | | | | |

**Fig. S1** Receiver operating characteristic curves of the weighted and unweighted screening scores for CDC/AAP severe periodontitis^30^


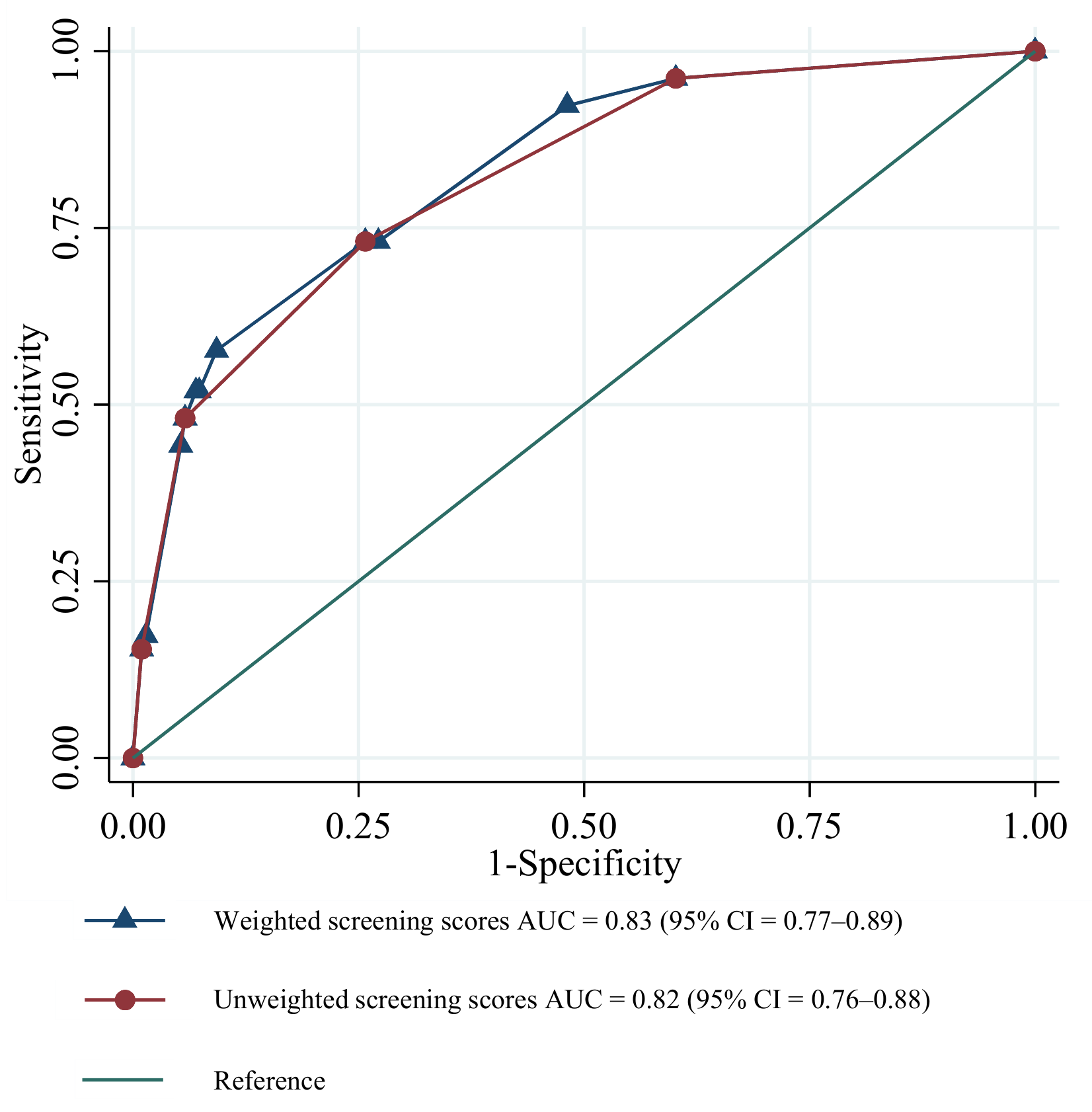


AAP, American Academy of Periodontology; CDC, Centers for Disease Control and Prevention
