# Supplementary figures and images for: Validation of a self-report questionnaire for periodontitis in a Japanese population
Source: Sci Rep. 2021 Jul 23;11:15078. doi: 10.1038/s41598-021-93965-4 (PMC8302714; doi:10.1038/s41598-021-93965-4)

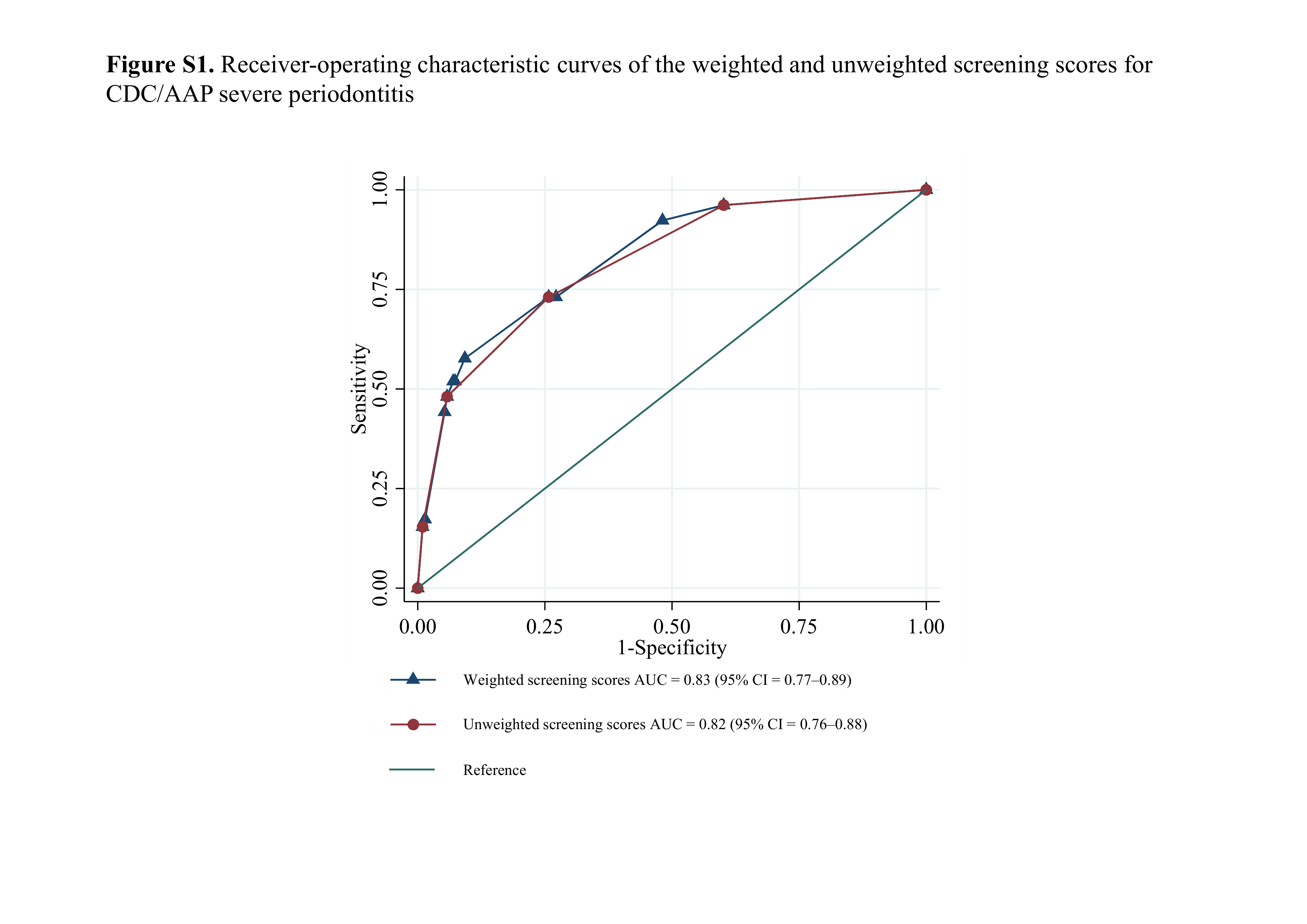

Supplement: Supplementary file 2 — Supplementary Information 2. [file 41598_2021_93965_MOESM2_ESM.tif]
